# Your Heart Function Has Normalized—What Next After TRED-HF?

**DOI:** 10.1007/s11897-023-00636-8

**Published:** 2023-11-24

**Authors:** Alexandros Kasiakogias, Aaraby Ragavan, Brian P. Halliday

**Affiliations:** 1 Inherited Cardiac Conditions Care Group, Royal Brompton and Harefield Hospitals, Part of Guy’s and St Thomas’ NHS Foundation Trust, London, UK; 2 National Heart and Lung Institute, Imperial College London, London, UK

**Keywords:** Heart failure, Improved ejection fraction, Recovered ejection fraction, Dilated cardiomyopathy, Drug withdrawal

## Abstract

**Purpose of Review:**

With the widespread implementation of contemporary disease-modifying heart failure therapy, the rates of normalization of ejection fraction are continuously increasing. The TRED-HF trial confirmed that heart failure remission rather than complete recovery is typical in patients with dilated cardiomyopathy who respond to therapy. The present review outlines key points related to the management and knowledge gaps of this growing patient group, focusing on patients with non-ischaemic dilated cardiomyopathy.

**Recent Findings:**

There is substantial heterogeneity among patients with normalized ejection fraction. The specific etiology is likely to affect the outcome, although a multiple-hit phenotype is frequent and may not be identified without comprehensive characterization. A monogenic or polygenic genetic susceptibility is common. Ongoing pathophysiological processes may be unraveled with advanced cardiac imaging, biomarkers, multi-omics, and machine learning technologies. There are limited studies that have investigated the withdrawal of specific heart failure therapies in these patients. Diuretics may be safely withdrawn if there is no evidence of congestion, while continued therapy with at least some disease-modifying therapy is likely to be required to reduce myocardial workload and sustain remission for the vast majority.

**Summary:**

Understanding the underlying disease mechanisms of patients with normalized ejection fraction is crucial in identifying markers of myocardial relapse and guiding individualized therapy in the future. Ongoing clinical trials should inform personalized approaches to therapy.

## Introduction

The goals of therapy for heart failure (HF) with reduced ejection fraction (HFrEF) include alleviation of symptoms and improvements in mortality and cardiovascular outcomes [1, 2]. With the advent of effective disease-modifying therapies, left ventricular reverse remodeling (LVRR) is currently observed in at least 40% of patients with non-ischemic dilated cardiomyopathy (DCM) and is associated with improved prognosis [3]. Indeed, the trajectory of the left ventricular ejection fraction (LVEF) offers important insight into the clinical course and outcome of patients with HF [4]. A number of pivotal investigations in the last decade have shed light on the clinical profile and outcome of patients with previously reduced LVEF that improves to > 40%. Interest in this patient group has driven the creation of new subcategories of HF (Table 1). There is currently an agreement that HF with improved EF (HFimpEF) includes patients with a previous LVEF ≤ 40% that increases to > 40%, typically with the contribution of guideline-directed medical therapy (GDMT) and device therapy [3, 5].

**Table 1:** Running/proposed definitions related to improvement in ejection fraction in patients with previous heart failure with reduced ejection fraction

**Left ventricular reverse remodeling:** The process of progressive restoration of cardiomyocyte size and left ventricular chamber geometry resulting in a leftward shift of the end-diastolic pressure–volume relationship toward normal values. Involved mechanisms include changes at cellular, interstitial, gene, structural–functional, hemodynamic, and neurohormonal levels
**Heart failure with improved ejection fraction (HFimpEF):** Patients with previous heart failure with reduced ejection fraction (LVEF < 40%) who experienced partial (40% ≤ LVEF < 50%) or complete (LVEF ≥ 50%) improvement in the LVEF, usually with a concurrent reduction in LV size, irrespective of the presence of symptoms or natriuretic peptide levels - **AHA/ACC/HFSA, 2022:** Previous LVEF ≤ 40% and a follow-up measurement of LVEF > 40% - **ESC/HFA, 2021:** History of LVEF ≤ 40% and later presentation with LVEF ≥ 50% - **Universal definition of heart failure, 2021:** HF with a baseline LVEF ≤ 40%, a ≥ 10-point increase from baseline LVEF and a second measurement of LVEF > 40%.* - **JACC scientific expert panel, 2020:** Documentation of an LVEF < 40% at baseline, ≥ 10% absolute improvement in LVEF and a second measurement of LVEF > 40%
**Heart failure with reduced ejection fraction in remission (HFrEFrem):** Previous LVEF of < 40% with subsequent increase to ≥ 50%, normalization of LV end-diastolic volume and NT-proBNP levels, and no HF symptoms, evidence of congestion or need for diuretic therapy
**Heart failure with permanently recovered ejection fraction (HFrecEF):** Absence of latent disease activity with permanent resolution of symptoms and signs of HF, normalization of LVEF (> 50%), and restoration of normal cardiac structure, function, biomarkers, energetics, and physiologic reserve.**
**Heart failure relapse:** Patients with HFrEFrem presenting with one or more of the following (with or without withdrawal of GDMT): (a) an absolute reduction in LVEF by more than 10% and to less than 50%, (b) an increase in LVEDV by more than 10% and to higher than the normal range, (c) a two-fold rise in baseline NT-proBNP concentration and to more than 400 ng/L, and (d) clinical evidence of heart failure.***

*LVEF* left ventricular ejection fraction, *GDMT* guideline-directed medical therapy, *NT-proBNP* NT-pro-brain natriuretic peptide

*An LVEF change > 10% minimizes misclassification due to inter-/intra-observer variability

**The term “recovered EF” was previously used interchangeably with “improved EF” to describe partial or complete normalization of LVEF irrespective of symptoms. It is suggested that it is reserved for asymptomatic patients with normalized LVEF and resolution of disease

***Definition derived from the TRED-HF trial

In a subset of asymptomatic patients with HFimpEF, the LVEF and natriuretic peptides return to normal, and it has been a clinical and research conundrum whether this represents true HF recovery or remission (HFrEF in remission, HFrEFrem). The TRED-HF (therapy withdrawal in recovered DCM) trial demonstrated that complete withdrawal of pharmacological therapy is associated with relapse of HF in a substantial proportion of patients with normalized LVEF [6]. Nevertheless, the underlying biology, clinical course, and outcomes of patients with LVEF improvement/normalization are incompletely understood. The current review provides an update on issues related to the management of the subgroup of patients with normalized LVEF focusing on dilated cardiomyopathy (DCM).

## The Continuum of Improving LVEF: Understanding the LVEF Trajectory

Ventricular dilation and reduction in LVEF are the principal features of DCM [7]. Left ventricular reverse remodeling involves an increase in LVEF, reflecting improvements in myocyte contractility, accompanied by regression of eccentric hypertrophy and fibrosis [8]. Factors that have been traditionally associated with LVRR include female sex, younger age, milder HF, shorter duration of the disease, and fewer comorbidities [9–11]. LVEF recovery may be partial (with an LVEF increasing to between 40 and 49%) or complete (normalized LVEF to ≥ 50%).

While 20–40% of patients are expected to transition to higher LVEF in just a few months, [9, 10, 12–14] evidence of LVRR may be observed as late as 2 years after initiation of therapy [15•]. An analysis of 4942 patients of the Swedish Heart Failure Registry revealed that 26% of patients with HFrEF at baseline showed an increase in LVEF to > 40% (with 10% to LVEF > 50%) and 25% of patients with HF with mildly reduced LVEF showed an increase in LVEF to > 50% [13•]. The exact rates of recovery are likely underestimated, considering that most relevant studies are based on a single repeat evaluation of LVEF. They are also likely to be greater now with the widespread implementation of highly effective contemporary GDMT and the use of medical devices [9, 10, 12, 14, 15

There are several key points to consider regarding patients with HFimpEF. Firstly, improvement or even normalization of LVEF does not guarantee the resolution of symptoms or return of biomarkers, such as natriuretic peptides, to normal [12, 12–15].

Secondly, the LVEF may deteriorate again, further supporting that in a substantial proportion of patients with normalized LVEF, there is a state of remission (HFrEFrem) rather than true permanent recovery [5, 6, 15–17]. Taken together, it appears that LVEF normalization does not reflect a return to the normal state but the transition to a lesser state of disease [3]. This two-way LVEF trajectory may depend on factors such as the root cause, disease duration, adherence to GDMT, or renewed exposure to cardiotoxicity or external stressors. In a retrospective analysis of 800 patients with non-ischaemic cardiomyopathy from the Trieste Heart Muscle Registry, an improved LVEF (LVEF > 40%) was documented in a remarkable 57% of the population over a median of 11-year follow-up period [15•]. The HFimpEF group was further divided into those with persistent and those with transiently improved LVEF. The latter represented 41% of the HFimpEF population, and the median time to relapse was 5 years despite continued medical therapy. Older age, lower LVEF, and longer disease duration at the time of improvement were associated with a greater risk of relapse. Nevertheless, the determinants of the duration of remission and the risk of recurrent LVEF drop have not been completely clarified.

Lastly, the prognosis of patients with improvements in LVEF continues to be investigated. Patients with HFimpEF have a better prognosis compared to those with persistent HFrEF and the degree of LVRR is associated with outcome [12–14, 16, 18]. The clinical course of HFimpEF appears distinct from that of HF with mildly reduced and preserved LVEF, despite the LVEF overlap [12, 18, 19]. Nevertheless, the overall survival appears worse than that of healthy controls and a proportion of patients experience adverse events in the long term, including heart failure hospitalizations and the need for advanced heart failure therapies. Current data are limited, and reliable predictors of recurrent HF remain unclear.

## The TRED-HF Study: Key Points

The TRED-HF study was an open-label, pilot randomized controlled study with a single-arm crossover phase examining the effect of phased and complete withdrawal of pharmacological therapy in asymptomatic patients with DCM and normalized LVEF over a 6-month period [6]. It sought to answer the question of whether stopping all HF medications was safe once LVEF has normalized, and signs and symptoms of HF had resolved. In other words, it investigated whether this represented “complete” or “apparent” healing. The study included 51 patients with a mean age of 55 years (67% men) and previously reduced LVEF (≤ 40%, median LVEF of 25%), presenting with an LVEF of ≥ 50% along with normal LV end-diastolic volume at enrolment. The absence of symptoms and normal NT-proBNP levels confirmed disease remission as per current definitions. A stepwise reduction in medications was performed over 16 weeks. During the randomized phase, 44% of the patients who were assigned to medication withdrawal relapsed compared to none in the control group. Importantly, 26% relapsed within just 8 weeks of taking their last medication. When medications were withdrawn in the control group in the follow-on phase, a further 36% of patients relapsed. An adequate response to restarting therapy was observed in most patients, with an LVEF of > 50% documented in 85% of patients at the subsequent clinic visit.

**Fig. 1:**
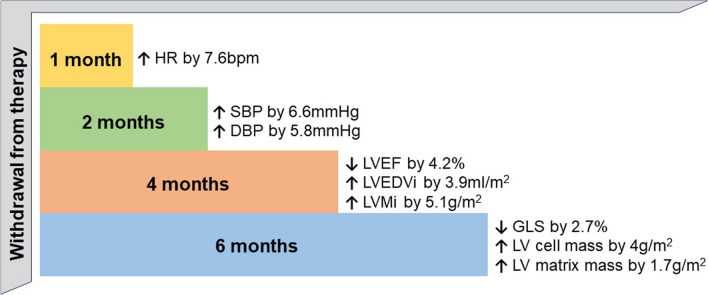
Main changes in clinical and imaging parameters observed in patients who withdrew from therapy in TRED-HF based on available measurements. HR heart rate, SBP systolic blood pressure, DBP diastolic blood pressure, LVEF left ventricular ejection fraction, LVEDVi left ventricular end-diastolic volume index, LVMi left ventricular mass index, GLS global longitudinal strain

The TRED-HF trial, despite being a small pilot study, provided important insight into a previously unknown territory in the field of HF. The results confirmed that a significant proportion of patients are in remission and that full withdrawal of medical therapy should not routinely be performed. The study also highlighted that it is important to identify discriminators of sustained recovery as well as early markers of relapse. A normal LVEF and an asymptomatic status provide an incomplete assessment of disease status and the risk of recurrent HF. Interestingly, in participants who withdrew from therapy, there was evidence of an increase in the heart rate and blood pressure in the first few weeks after withdrawal, in keeping with neurohormonal (re-)activation and increased myocardial work. Patients who relapsed had a 10 beats/min greater rise in heart rate compared to those who did not relapse [20•]. Heart rate and heart rate change were associated with relapse. Imaging evidence of LV remodeling was evident from as early as 4 months after withdrawal (Fig. 1) [21, 22]. In those who relapsed, changes in LVEF preceded changes in NT-proBNP, suggesting that natriuretic peptides may not be good markers of early relapse.

## How Can Disease Etiology and Underlying Disease Activity Inform Decision-Making?

Dilated cardiomyopathy results from a wide range of genetic and acquired causes [7]. An interaction between genetic predisposition and environmental factors may apply in a significant proportion of patients (“seed and soil” hypothesis, first used by Stephen Paget in 1889 to describe cancer metastasis) [5]. The underlying processes contributing to disease likely impact the rate and degree of LVRR and the future risk of relapse. In any given HFimpEF patient, the genetic susceptibility, repeat exposure to an external myocardial insult or stress and the effects of non-cardiac comorbidities should be taken into account. Predicting which patients with normalized LVEF are at increased risk of relapse (particularly after withdrawal of cardiac medications) appears to be a complex process. Several steps that need to be addressed in this context are outlined below.

### Revisiting Specific Triggers of Cardiomyopathy

**Fig. 2:**
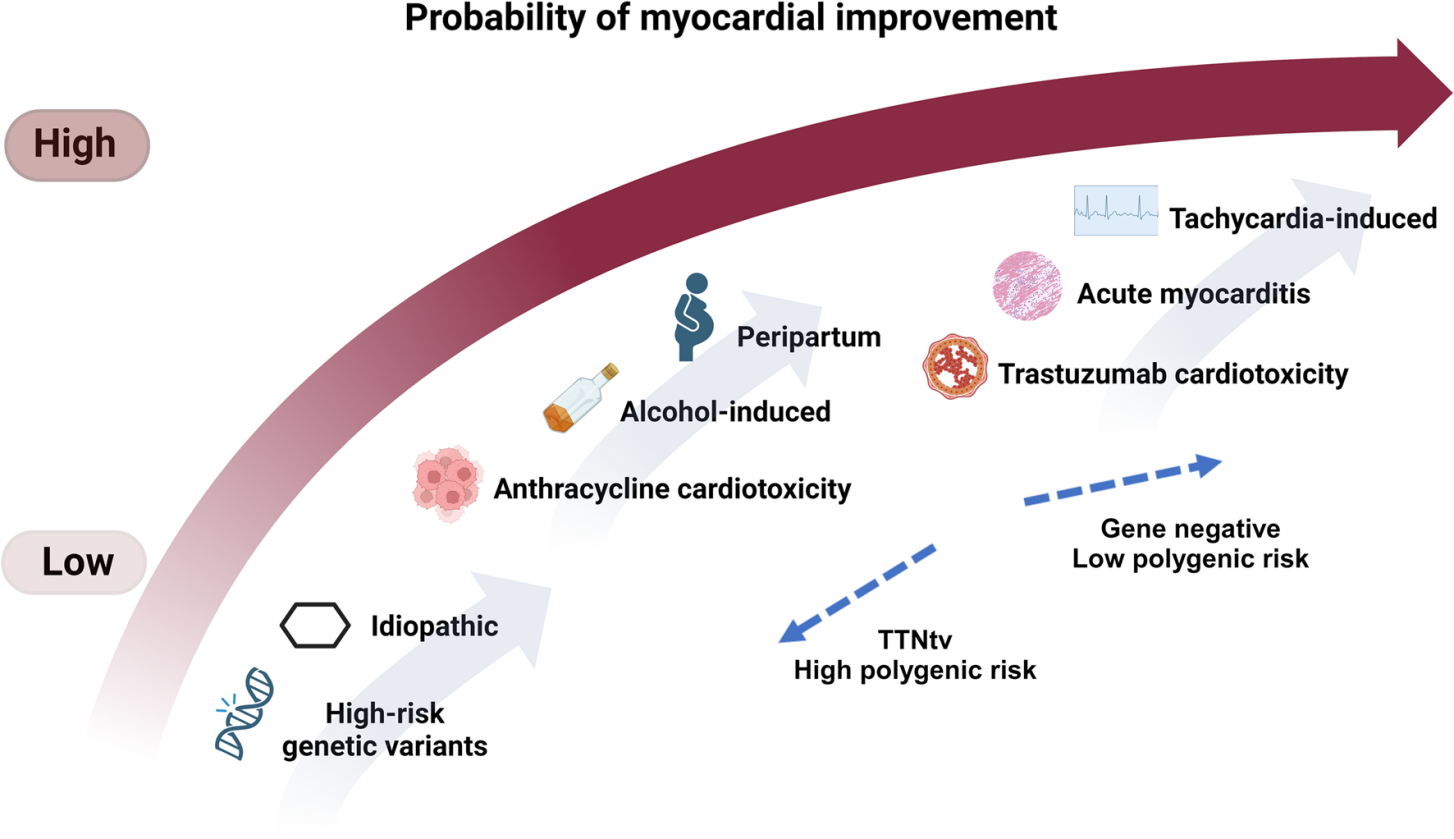
Suggested probability of LVEF improvement and normalization among different common aetiologies of dilated cardiomyopathy. Increasing evidence suggests that the underlying genetic background influences myocardial susceptibility to the respective trigger as well as clinical course and recovery. Other factors, including the presence/extent of fibrosis and co-morbidities, further affect myocardial response (spontaneous or following therapy). It is acknowledged that there is insufficient data comparing the clinical course of the various causes of dilated cardiomyopathy. The present figure may, therefore, require revision as new information emerges. TTNtv truncating variant in titin. (Created with Biorender.com)

It has traditionally been considered that once an inciting factor of DCM is completely removed, the probability of satisfactory to full recovery is high [3]. Conditions such as tachycardia-induced, alcohol, and peripartum cardiomyopathy are typical examples. However, continuous advances have helped us understand that a dual or multiple-hit pathophysiology often applies. For instance, a rare genetic variant associated with DCM may be identified in a significant proportion of patients with alcohol cardiomyopathy, myocarditis, and anthracycline-induced cardiotoxicity [23, 24]. Polygenic risk resulting from common genetic variation is also likely to drive myocardial vulnerability [25]. Genetic variation affects the pathophysiological mechanisms related to the myocardial response to chemotherapeutic agents such as anthracyclines and trastuzumab [26, 27]. These observations may have implications in terms of targeted treatments and decisions regarding continuation of conventional therapy (Fig. 2). It may be tantalizing to withdraw medications in conditions attributed to a single resolved triggering factor, but this may not always be the case.

A representative example of multifactorial etiology is peripartum cardiomyopathy, a condition with current rates of LVEF improvement ranging from 20 to 80% [28]. Time to recovery is variable and may be relevant to future risk of relapse. A tangible risk of deterioration even in the absence of pregnancy has been described [29]. Peripartum cardiomyopathy is now considered a complex syndrome involving vascular, hormonal, and inflammatory/autoimmune processes [30, 31]. Mitochondrial dysfunction may also play an important role [32]. Importantly, a genetic contribution has been identified in several studies that show a prevalence of 10–15% of truncating variants in titin (TTN, TTNtv) as well as genes associated with arrhythmic phenotypes [33, 34].

### Genetic Cardiomyopathies

Genetic profiling informs the chances of observing LVRR and may contribute to decision-making for patients with HFimpEF/HFrEFrem [35–38]. Variants in genes of the nuclear envelope and cytoskeleton proteins are associated with lower rates of LVRR [36, 38, 39]. TTNtv are the most common variants in DCM and are often associated with a mild disease course and high rates of LVRR [39–41]. Whether the prevalence of rare variants in cardiomyopathy genes or the polygenic risk differs in patients with HFrEFrem compared to those with persistent HFrEF is a matter of investigation.

Recent data support gene-specific mechanisms of HF recurrence [42, 43]. For instance, in a retrospective analysis of 239 patients with TTNtv from the Maastricht DCM registry, both patient groups with and without TTNtv showed a similar sharp increase in LVEF at up to 2 years and similar rates of LVRR [42]. Subsequently, the LVEF slowly declined in those with TTNtv but remained stable in the non-TTNtv group. It has been suggested the ability of the heart to sustain energy demands may become less effective over time and this may be more marked in carriers of TTNtv [44]. Such results set the scene for further investigations with respect to myocardial adaptations in different genotypes. A better understanding of energetic and mechanical changes may identify the substrate for relapse in the presence of hemodynamic stressors.

### Ongoing Disease Processes and the Role of Cardiac Imaging and Biomarkers

**Fig. 3:**
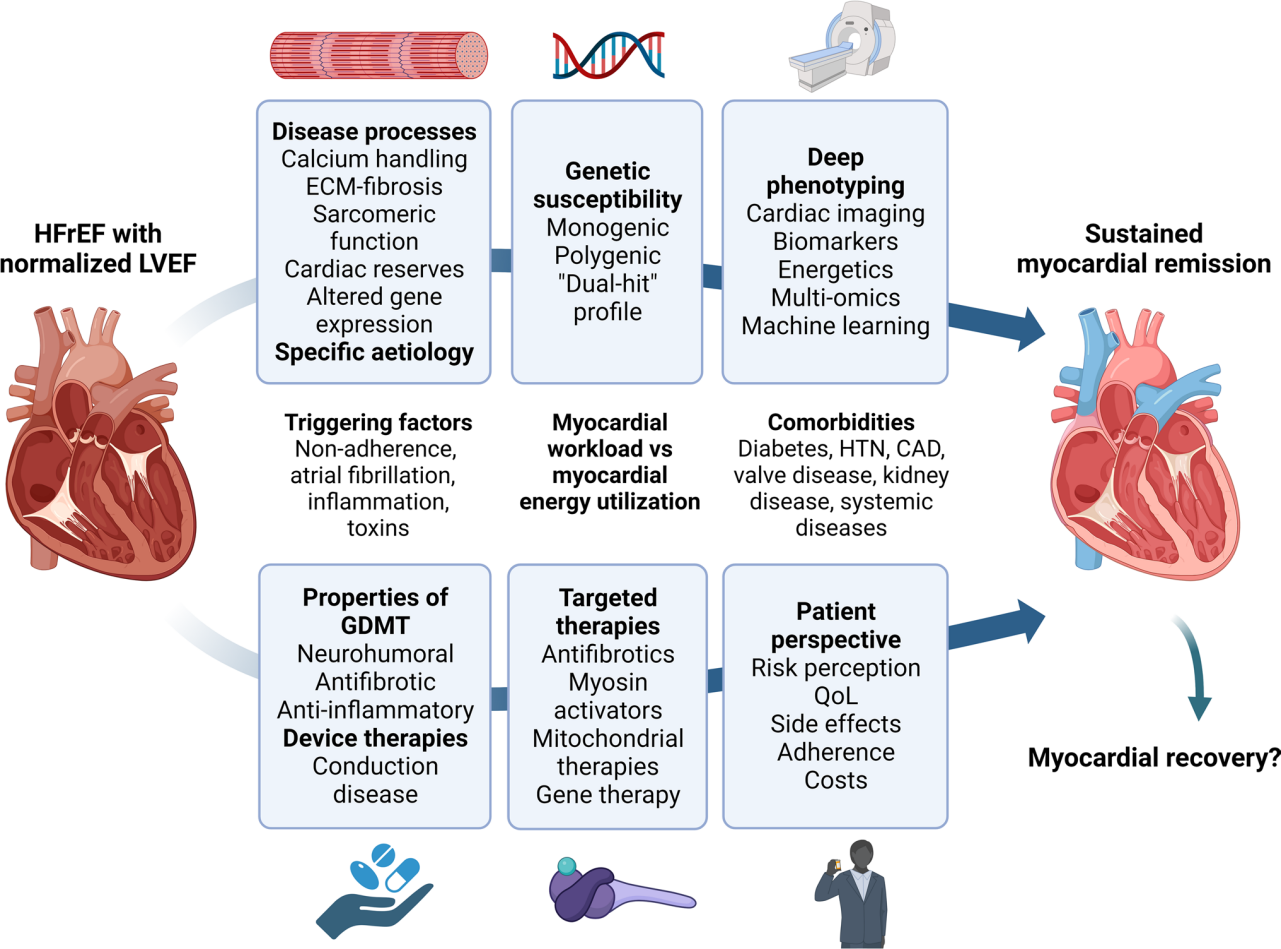
Proposed pathway to applying precision medicine principles in heart failure patients in myocardial remission to reduce the risk of relapse. A combination of our understanding of the ongoing substrate of disease and the role of existing and upcoming therapies in targeting disease mechanisms is central to providing personalized care. HFrEF heart failure with reduced ejection fraction, LVEF left ventricular ejection fraction, ECM extracellular matrix, HTN hypertension, CAD coronary artery disease, GDMT guideline-directed medical therapy, QoL quality of life. (Created with Biorender.com)

Data from the Penn Heart Study, where myocardial recovery was defined using an LVEF cut-off of > 50%, demonstrated persistent biomarker evidence of inflammation, neurohormonal activation, and myocardial injury in this population [12]. Combining data from clinical assessment as well as advanced imaging and biomarkers may enable the characterization of ongoing disease processes and prediction of the risk of relapse (Fig. 3) [4, 45].

Imaging data beyond LVEF may inform management. Global longitudinal strain has been shown to vary widely, denoting large heterogeneity of intrinsic myocardial function [46–49]. A significant proportion of HFimpEF patients have reduced GLS despite a normal LVEF, and this has been associated with a greater risk of relapse and worse outcomes [46–50]. A study in 206 patients with non-ischaemic cardiomyopathy and normalized LVEF (LVEF > 50%) showed that a GLS of < 16% was associated with a threefold increased risk of mortality and major adverse cardiac events [47•]. A recent retrospective study in 699 patients reported that left atrial reverse remodeling, defined as > 15% reduction in the left atrial end-systolic volume, was observed in ~ 60% of patients with improved LVEF and was associated with a lower risk of cardiovascular death and hospitalization [51]. In a small study of 96 patients with HFimpEF, diastolic dysfunction, defined as an elevated E/E′ (> 12.1), was associated with a higher risk of a similar endpoint [52].

The availability of cardiac magnetic resonance (CMR) has been increasing and CMR is recommended for assessment of DCM in current guidelines [1]. Evaluation for myocardial fibrosis by CMR provides important prognostic information in DCM [53, 54]. Evidence suggests that replacement myocardial fibrosis predicts lower rates of LVRR [55]. A retrospective review of 148 patients with recent-onset DCM showed that one-third of the population with initially improved LVEF (≥ 45%) presented with re-worsening of their LVEF which was associated with the degree of fibrosis on CMR [56]. Preliminary data from TRED-HF showed that there was expansion of the extracellular matrix during treatment withdrawal, suggestive of ongoing fibrotic activity [22].

Other domains of cardiac phenotyping are continuously evolving [57]. A number of omics technologies are available that can contribute to the biological characterization of these patients and various computational methods are being investigated for the utilization of large amounts of data to identify pathways of disease [58–60]. Techniques such as MR spectroscopy may further contribute to our understanding of ongoing energy deficits in these patients [61]. Molecular identification of fibrotic activity is further achieved with the use of collagen biomarkers and dedicated radiotracers, while therapies targeting fibrosis are concurrently under development [62–64].

### Phenogrouping for Targeted Therapies

Mining of large datasets using machine-learning technologies is a novel method of grouping patients who share common features; these may reflect separate biologies and mechanisms of disease and may allow the use of targeted therapies [65–67]. In a study of 426 patients with DCM, clustering of clinical, imaging, genetic, and proteomic data identified three distinct DCM subgroups, including a profibrotic metabolic subtype characterized by extensive fibrosis, and increased prevalence of diabetes and kidney disease [66•]. Importantly, regression analysis identified a simple 5-variable model to assign patients to the relevant group. Similar investigations in patients with HFimpEF/HFrEFrem are limited. One study in 889 patients with improved LVEF to > 50% utilized an unsupervised clustering algorithm to group patients based on differences in 11 pre-defined variables [68]. The study identified 7 phenotypes of HFimpEF, signifying marked heterogeneity. In addition, one of the phenotypes was associated with an increased risk of relapse and greater mortality. This was a retrospective analysis of an administrative dataset, and the study results were not externally validated. Nevertheless, they set an example of how such methodologies may identify patient groups that benefit from different therapeutic strategies.

## The Role of Current Disease-Modifying Therapies. What Is the Evidence?

Most HF trials in patients with an LVEF > 40% excluded patients with previous HFrEF, and no such trials have included asymptomatic patients with improved LVEF. The optimal HF drug regimen for these patients remains unknown. There is currently an agreement that GDMT should be maintained at maximal tolerated doses in patients with continued symptoms and signs of HF, irrespective of the LVEF [2, 3]. The TRED-HF trial showed that withdrawal of all HF medications in patients with HFrEFrem was associated with a high rate of relapse [6]. In this trial, all patients were on a renin-angiotensin-system (RAS) blocker and most on a beta-blocker. Less than half were on a mineralocorticoid receptor antagonist (MRA) and 12% were on a diuretic. No patients were on angiotensin-receptor neprilysin inhibitors (ARNi) or sodium-glucose cotransporter 2 inhibitors (SGTLT2i). Based on the results of the TRED-HF trial, the most recent iteration of the AHA/ACC/HFSA guidelines recommended that GDMT should be continued to prevent HF relapse even in asymptomatic patients with HFimpEF [2]. The recent ESC/HFA guidelines also recommend continued treatment [1]. It remains unclear whether GDMT should continue at the maximum dose or whether there may be a “simplified” regimen that can effectively maintain remission in specific subgroups.

Management of neurohormonal dysregulation and myocardial stress are crucial aspects of therapy [1]. The activity of traditional pathophysiological HF mechanisms in HFrEFrem patients is unclear and likely varies between patients as discussed above. The patient profiles and circumstances that allow the safe reduction and/or withdrawal of agents in asymptomatic patients with HFrEFrem are currently being scrutinized.

### Loop Diuretics

It appears reasonable to reduce or stop loop diuretics in patients without evidence of congestion [69]. The ReBIC-1 (Rede Brasileira de Estudos em Insuficiência Cardíaca) trial confirmed that diuretic withdrawal is safe in stable HF [70]. The study enrolled 188 patients of a mean age of 59 years and with an LVEF ≤ 45% (mean LVEF 32%) who were on optimized HF treatment including low-dose loop diuretics. The participants were in NYHA class I or II, without congestion, and free of recent HF admissions. Diuretic withdrawal versus continued administration was not associated with worse self-reported dyspnoea or need for furosemide use during the 90-day period of the study.

### Beta-Blockers

An analysis of the TRED-HF trial showed that rises in heart rate preceded overt myocardial dysfunction in patients who relapsed following medication withdrawal [20•]. It is reasonable to speculate that continued suppression of the sympathetic nervous system may be required to control myocardial work and stress and avoid HF recurrence. Older studies performed over 20 years ago had shown that withdrawal of beta-blockers was associated with worsening of LV function in patients with congestive HF and dilated cardiomyopathy respectively [71–73]. A retrospective analysis utilizing data from a national Japanese database investigated the association of beta-blocker use with myocardial relapse in HFimpEF patients with a current LVEF ≥ 40% [74]. Propensity score matching yielded a total of 1087 patient pairs (on and off beta-blocker therapy). The study showed that beta-blocker use was associated with a 23% lower risk of a decrease in LVEF ≥ 10% at 2 years of follow-up. These results suggest that beta-blockers may be an important pillar of maintaining remission.

### Renin-Angiotensin Blockers and Angiotensin-Receptor Neprilysin Inhibitors

Inhibition of the RAS system with RAS blockers has a pivotal role in the treatment of HFrEF and withdrawal of therapy is associated with worsening symptoms and ventricular function [75]. As mentioned, all patients in TRED-HF were on a RAS blocker; however, the exact contribution of RAS inhibition to sustaining remission is unclear. ARNi provides prognostic benefits for patients with HF across the entire range of LVEF [76]. Updated analyses of the PARAGON-HF (Prospective Comparison of ARNi with ARB Global Outcomes in HF with Preserved Ejection Fraction) trial showed significant reductions in HF events in patients with LVEF > 45%, although, the benefit was more evident in patients with an LVEF at the lower end of the spectrum. Notably, it excluded patients with a previous LVEF of less than 40% [77]. There is no data investigating whether continuation or initiation of ARNi sustains remission more effectively in asymptomatic patients with normalized LVEF. Switching to a RAS blocker when there is evidence of remission may be an attractive strategy, considering the cost limitations for some insurance systems.

### Mineralocorticoid Receptor Antagonists

The added benefit of MRA on LVRR in mild to moderately symptomatic patients with HF on appropriate background therapy has been questioned [78]. Whether ΜRAs need to be continued once the LVEF has normalized is unclear. In TRED-HF, of the 15 patients who were taking MRA at the start of the study and subsequently relapsed, 10 did not restart an MRA after the study and all managed to achieve remission again [6].

An open-label, controlled, prospective observational study from China examined the effects of withdrawal of spironolactone in 70 asymptomatic patients with idiopathic DCM and HFimpEF with an LVEF > 40% [79]. This was not randomized and spironolactone withdrawal versus continuation was based on an informed decision by the patient. The mean LVEF was 46% and NT-proBNP levels ranged from 298 to 1793 pg/L. The primary endpoint of myocardial relapse (defined as at least one of a > 10% LVEF reduction or > 15% LVESVi or a twofold rise in NT-proBNP concentration or clinical signs and symptoms of HF) was observed in 58% of patients in the withdrawal group and 13% in the continuation group over 12 months. Interestingly, 74% of patients who relapsed had clinical evidence of HF without reduction in LVEF. Νo deaths or adverse cardiovascular events were reported. The study should be interpreted in the context of a non-randomized design and a population of patients with ongoing evidence of HF.

### Sodium-Glucose Cotransporter 2 Inhibitors

Similar to ARNi, large trials have shown that SGLT2i reduces cardiovascular death and HF admissions in symptomatic patients with HF across the entire range of LVEF [80]. Specifically, the DELIVER trial (Dapagliflozin Evaluation to Improve the Lives of Patients with Preserved Ejection Fraction) investigated the prognostic benefit of dapagliflozin in patients with an LVEF of more than 40% and elevated natriuretic peptides [81]. The study enrolled 6263 patients with a mean age of 72 years, of which most were in NYHA class II–III. The study was unique in that it allowed for patients with a previous LVEF of < 40% to be included. In particular, 18% of patients fulfilled the criteria for HFimpEF [82]. The study showed an 18% reduction in cardiovascular death and worsening HF that appeared consistent in the HFimpEF group [81, 83, 84]. On the other hand, SGLT2i exhibits a number of properties (e.g., antifibrotic effects, metabolic modulation) that may target pathways associated with myocardial relapse [85].

## Should Device Therapy Be Continued?

The benefit of ICDs in patients with DCM is currently debated [86]. Given the high incidence of LVRR, many advocate a longer period of contemporary GDMT (e.g., up to 9 months) before considering an ICD for primary prevention, thus allowing enough time for myocardial recovery [87, 88]. There is limited information regarding the effectiveness of ICDs in the setting of LVEF normalization [89]. Indeed, a significant proportion of patients with DCM are young and face risks of serious device complications in the medium- and long-term. Available information supports that the arrhythmic risk is reduced but not removed when LVEF improves [90, 91]. There is, therefore, a general consensus that generator change should be performed in patients with HFimpEF [3]. The presence of high-risk genetic variants, the extent of myocardial fibrosis on CMR, GLS, cardiac biomarkers such as natriuretic peptides and ST2 may identify those patients least likely to reverse remodel and most likely to benefit from an ICD [1, 38, 53, 92–94].

Cardiac resynchronization therapy (CRT) is associated with LVRR and improved outcomes, including lower rates of ventricular arrhythmias [95]. It is indicated in the presence of ventricular dyssynchrony assessed primarily by electrocardiography and there is consensus that it should be continued once LVEF recovers due to a high risk of recurrent HF in withdrawal studies [3, 96]. The STOP-CRT trial further investigated whether withdrawing neurohumoral blockade (RAS blockers and/or beta-blockers) is feasible in patients who respond well to CRT presenting with normalized LVEF [97•]. The rate of relapse was low suggesting that in these patients, drug withdrawal may be safe and feasible.

## Involving the Patient and Shared Decision-Making

Heart failure patients perceive good quality of life as a major scope of their clinical management [98, 99]. Patients are often keen to explore the option of having their medications reduced (in terms of dose and/or quantity) despite the possible risk of relapse. Patients are often of young age and may not wish to continue taking medications long-term if there is no strong evidence of benefit [6]. Some are concerned about medication-related side effects which may affect quality of life [100]. For women who intend to become pregnant, discontinuing ACEi/ARB/ARNi, MRA, and SGLT2i is indicated. In addition, reducing the number of medications may provide financial relief depending on patient contribution by country. This could be the case when switching from an ARNi to an ACEi, should the risk of HF morbidity remain similar. Patient preference should be considered also with respect to adherence to complex medication regimens.

## Unanswered Questions and Future Research

The TRED-HF study provided randomized data to support the continuation of at least some therapy in patients with HFrEFrem. However, several questions remain. Uncovering predictors of myocardial relapse will be key for the management of this patient group (Table 2). The optimal medical HF regimen for patients with HFrEFrem to reduce the risk of HF recurrence remains unclear. Appropriate patient phenotyping is required for a personalized approach. Understanding the underlying pathophysiology in an individual patient may help guide streamlined therapies targeting individual mechanisms of relapse. The TRED-HF 2 trial is an open-label randomized trial that will examine whether it is safe and feasible to withdraw MRA and/or SGLT2i in patients with DCM and HFrEFrem who are on background therapy with a RAS blocker and beta-blocker. The main outcome will be HF relapse while imaging and biomarker information will be acquired during the study. The study protocol of another randomized study has been published that will investigate whether halving neurohormonal blockers (RAS blockers or ARNi and beta-blockers) is non-inferior to the original dose in terms of protection from HF relapse/hospitalization [101].

**Table 2:** Unresolved/unclear questions regarding the management of asymptomatic patients with normalized LVEF

• Which DCM phenotypes are more likely to be associated with myocardial remission and recovery?
• What are the markers of increased risk of myocardial relapse (with/without withdrawal of HF medications)?
• What are the early indices of HF relapse before overt clinical decompensation that would in turn guide early drug optimization?
• Is reducing the dose/quantity of HF medications feasible without increasing the risk of myocardial relapse and cardiac events and does this improve quality of life? Are there “pillars of therapy for HFrEFrem”?
• What is the best strategy to reduce/withdraw drugs with respect to safety and clinical stability?
• Is restarting therapy following relapse associated with a similar incidence of recovery?
• What is the optimal framework of deep phenotyping to provide personalized treatment?
• Which current/future therapies may be effective in targeting mechanisms sustaining myocardial remission and potentially achieving complete recovery?
• What is the best way to make a shared decision about the need for continued heart failure therapy?

*LVEF* left ventricular ejection fraction, *DCM* dilated cardiomyopathy, *HF* heart failure

## Conclusions

Management of patients with HFrEFrem will be an increasingly common scenario. Clarifying the underlying disease processes and the best clinical management of these patients must be a major priority. The accumulating evidence suggests that this newly defined HF category should be approached separately from other HF classes of (near-) normal LVEF associated with ongoing evidence of the clinical HF syndrome. The TRED-HF trial confirmed that these patients are often in remission while the path to complete recovery is less clear. It is possible that not all drug components of contemporary GDMT are required to maintain remission and reduce the risk of cardiac events, but separate patient profiles may benefit from distinct and individualized medical regimens. This will, hopefully, be elucidated with ongoing clinical investigations.

## Abbreviations

ACC: American College of Cardiology
ACEi: Angiotensin-converting enzyme inhibitor
AHA: American Heart Association
ARB: Angiotensin receptor blocker
ARNi: Angiotensin-receptor neprilysin inhibitor
CMR: Cardiac magnetic resonance
CPET: Cardiopulmonary exercise test
CRT: Cardiac resynchronization therapy
DCM: Dilated cardiomyopathy
ESC: European Society of Cardiology
GDMT: Guideline-directed medical therapy
GLS: Global longitudinal strain
HFA: Heart Failure Association
HF: Heart failure
HFimpEF: Heart failure with improved ejection fraction
HFrEF: Heart failure with reduced ejection fraction
HFrEFrem: Heart failure with reduced ejection fraction in remission
HFSA: Heart Failure Society of America
ICD: Implantable cardioverter defibrillator
LVEDVi: LV end-diastolic volume index
LVEF: Left ventricular ejection fraction
LVESVi: Left ventricular end-systolic volume index
LVRR: Left ventricular reverse remodeling
MRA: Mineralocorticoid receptor antagonist
NT-proBNP: N-terminal pro-b-type natriuretic peptide
NYHA: New York Heart Association
RAS: Renin-angiotensin system
SGTLT2i: Sodium-glucose cotransporter 2 inhibitor
TTN: Titin
TTNtv: Truncating variant in titin
